# TLR2 stimulation induces cardiac inflammation but not cardiac depression *in vivo*

**DOI:** 10.1186/1476-9255-10-33

**Published:** 2013-10-30

**Authors:** Olaf Boehm, Pascal Knuefermann, Johannes Plueck, Markus Schwederski, Heidi Ehrentraut, Sied Kebir, Ralph Lohner, Markus Velten, Siegfried Morath, Alexander Koch, Kai Zacharowski, Christian Grohé, Andreas Hoeft, Georg Baumgarten, Rainer Meyer

**Affiliations:** 1 Department of Anesthesiology and Intensive Care Medicine, University Hospital Bonn, Sigmund-Freud-Str. 25, D-53105 Bonn, Germany; 2 Institute of Physiology II, University of Bonn, Nussallee 11, D-53115 Bonn, Germany; 3 Department for Pneumology, Evangelische Lungenklinik, Lindenberger Weg 27, D-13125 Berlin-Buch, Germany; 4 Clinic of Anesthesiology, Intensive Care and Pain Therapy, University Hospital Frankfurt, Theodor-Stein-Kai 7, D-60590 Frankfurt, Germany; 5 Joint Research Centre, Institute for Health and Consumer Protection, Via E. Fermi 2749, I-21027, Ispra, Italy

**Keywords:** LTA, TLR2, Sepsis, Cardiac dysfunction, Cardiac contractility

## Abstract

**Background:**

Bacteria such as *Staphylococcus aureus* induce myocardial dysfunction *in vivo.* To rectify conflicting evidence about the role of TLR2 signaling and cardiac dysfunction, we hypothesized that the specific TLR2 agonist purified lipoteichoic acid (LTA) from *S. aureus* contributes to cardiac dysfunction *in vitro* and *in vivo*.

**Methods:**

Wildtype (WT-) and TLR2-deficient (TLR2-D) mice were challenged with LTA and in comparison with equivalent doses of lipopolysaccharide (LPS) and CpG-oligodeoxynucleotide (CpG-ODN). TLR2-expression, NFκB as well as cytokine response were determined. Sarcomere shortening of isolated cardiomyocytes was analyzed *in vitro* and cardiac function *in vivo* after stimulation with LTA.

**Results:**

LTA induced up-regulation of TLR2 mRNA, activation of NFκB and cytokine expression within 2–6 h in WT-, but not in TLR2-D hearts. Cytokines were also elevated in the serum. LPS and CpG-ODN induced a more severe cardiac inflammation. *In vitro* incubation of cardiomyocytes with LTA reduced sarcomere shortening via NO at stimulation frequencies ≤ 8 Hz only in WT cells. However, hemodynamic parameters *in vivo* were not affected by LTA challenge.

**Conclusions:**

LTA induced cardiac inflammation was relatively weak and sarcomere shortening was reduced only below physiological heart rates. This may explain the apparent contradiction between the *in vivo* and *in vitro* LTA effects.

## Background

Impairment of cardiac function independently increases mortality during septic shock [1]. Experimental studies show a similar pattern of cardiac dysfunction in Gram-negative and Gram-positive sepsis [2]. Epidemiological data indicate increasing incidence of Gram-positive sepsis [3,4] pointing to a growing need to further elucidate mechanisms underlying cardiac dysfunction during Gram-positive sepsis.

An increase in tumor necrosis factor (TNF)-α and interleukin (IL)-1β contributes to cardiac dysfunction during sepsis in humans [5-7] and has been directly linked to Toll-like receptor (TLR) signaling. Of the eleven human TLRs described so far [8] TLR2 binds wall components from Gram-positive bacteria thereby inducing an inflammatory response. However, controversy still exists about the exact role of TLR2-dependent inflammation and the onset of cardiac dysfunction. On the one hand heat-inactivated *Staphylococcus aureus* (*S. aureus)* has been shown to induce cardiac TNF-α, IL-1β, and nitric oxide (NO) [9] the latter being another pivotal stepstone for the development of septic cardiac dysfunction and failure. More importantly, TLR2-deficient mice were protected against *S. aureus*-induced myocardial dysfunction and cytokine production [9]. However, recent studies of Plitas et al. and of our group have revealed a pivotal role of CD14/TLR9 signaling in polymicrobial sepsis [10,11]. In addition, TLR2 signaling appears to be of minor importance in vascular inflammation during polymicrobial sepsis [11]. These contradictory results justify further characterization of TLR2-dependent inflammation and its role in the development of cardiac dysfunction.

Therefore, we hypothesized that the bacterial cell wall component lipoteichoic acid (LTA), a specific TLR2 ligand, contributes to cardiac dysfunction. To test this hypothesis we applied highly purified LTA and monitored mediators of inflammation in the heart and serum, as well as physiological parameters representative for *in vitro* and *in vivo* cardiovascular function.

## Methods

### Animals

Male 12–14 week old C57BL/6 wild-type (WT) mice were purchased from Charles River (Charles River, Sulzfeld, Germany). TLR2-deficient (TLR2-D) mice were kindly provided by Prof. Shizuo Akira (Osaka University) and back-crossed onto a C57BL/6 background. Mice received water *ad libitum*, standard rodent chow and were housed in pathogen-free cages. The investigation conformed to the Guide for the Care and Use of Laboratory Animals published by the US National Institutes of Health (NIH Publication No. 85–23, revised 1996), and animal procedures were approved by the local committee for animal care (Bezirksregierung Köln, Cologne, Germany).

### Study design

**Figure 1:**
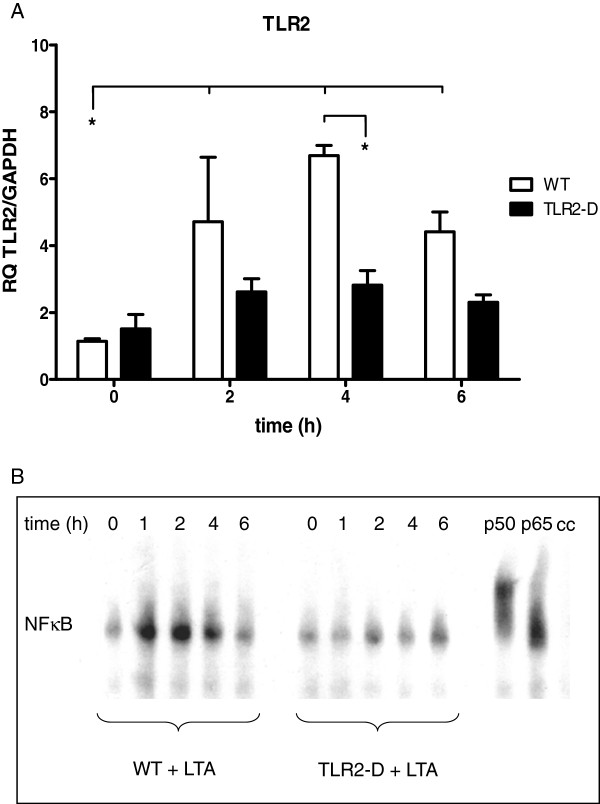
**Time course of inflammatory parameters in hearts of WT- and TLR2-D mice after LTA challenge (15 mg/kg, i.p). A**: RT-qPCR of TLR2. Peak expression was detected after 4 h. (*p < 0.05; n = 6; mean ± SEM). **B**: NFκB-DNA binding activity following LTA stimulation (EMSA). Lipoteichoic acid treatment activated NFκB in WT- but not in TLR2-D mice. The NFκB-complex mainly consisted of p50 and p65 as depicted by supershift assay (EMSA is representative of three animals per group).

**Figure 2:**
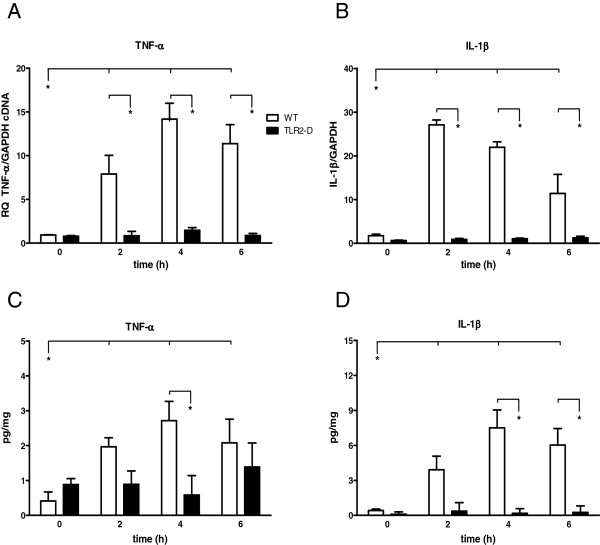
**Time course of pro-inflammatory cytokines in hearts of WT- and TLR2-D mice following LTA administration (15 mg/kg, i.p.). A, B**: RT-qPCR of TNF-α and IL-1β Both cytokines revealed a significant increase in WT-mice after 2 h (*p < 0.05; n = 6; mean ± SEM). **C, D**: ELISA of TNF-α and IL-1β. LTA administration led to a significant increase in protein expression of both TNF-α and IL-1β in WT- but not TLR2-D mice with a maximum at 4 h (*p < 0.05; n = 6; mean ± SEM).

**Figure 3:**
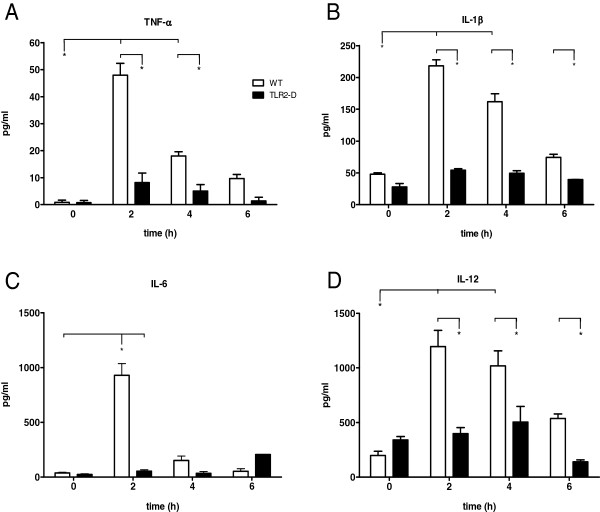
**Time course of serum cytokines in WT and TLR2-D mice after LTA stimulation (15 mg/kg i.p.). A-D**: Multiplex cytokine assay of TNF-α, IL-1β, IL-6 and IL-12. Peak protein levels of all cytokines were detected 2 h after stimulation in WT-mice. No increase was detected in TLR2-D animals (*p < 0.05 vs. TLR2-D; n = 5; mean ± SEM).

The study was conducted in four sets of experiments:

A. *Cardiac and systemic inflammation*: 0, 2, 4, and 6 h after i.p. injection of LTA (15 mg/kg) pattern recognition receptors (PRRs) and inflammatory mediators were monitored in the hearts and in the serum of WT and TLR2-D mice (Figures 1, 2, 3).

**Figure 4:**
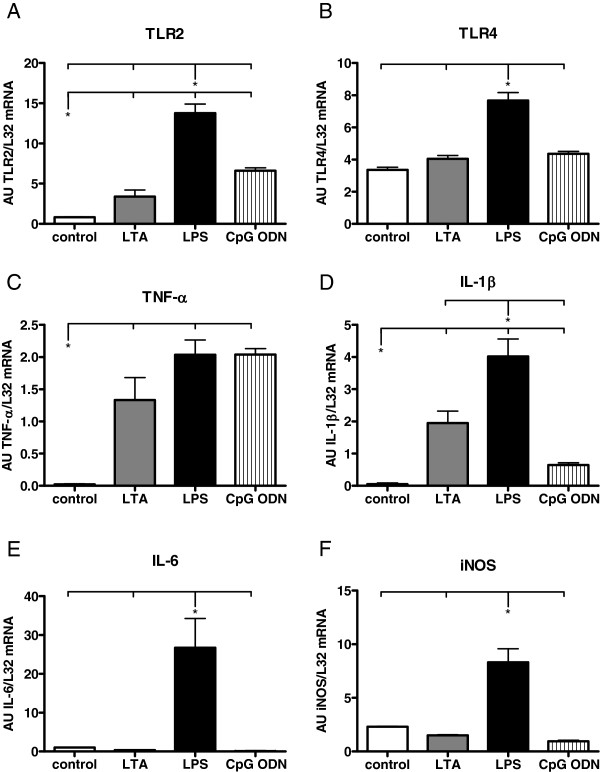
**PRR and cytokine mRNA expression in hearts of WT mice 4 h after stimulation with different TLR ligands as detected by RPA. B, C, D**: LTA stimulation (15 mg/kg, i.p) resulted in a significant increase of mRNA expression of TLR2, TNF-α and IL-1β. **A-F**: LPS stimulation (20 mg/kg, i.p.) led to a significant increase among all investigated parameters. **B, E**: CpG-ODN stimulation (1 nmol/g, i.p.) induced a significant increase of TNF-α and TLR2 expression (Values are expressed as arbitrary units, AU; p < 0.05; n = 4; mean ± SEM).

B. *Comparison of different TLR ligands*: 4 h after i.p. injection of LTA (15 mg/kg), LPS (20 mg/kg) or CpG-ODN 1668-thioate (1 nmol/g) mRNA expression of PRRs, inflammatory cytokines and iNOS was analyzed in the hearts of WT mice (Figure 4).

**Figure 5:**
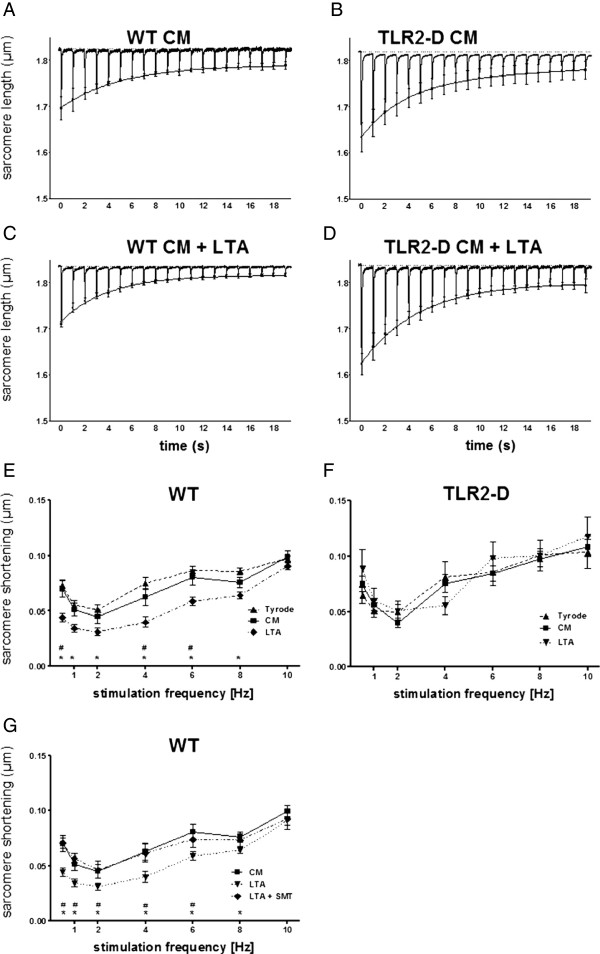
**Sacomere shortening of isolated cardiomyocytes after incubation with LTA (10 μg/ml). A-D**: Averaged original recordings of sarcomere shortening from WT- and TLR2-D cardiomyocytes with LTA or in culture medium (CM) alone. After 30 s stimulation-pause cells were stimulated at 1 Hz. The mean peak values (± SEM) of sarcomere shortenings were fitted by a double exponential function to give an impression of the time course of the staircase. Lipoteichoic acid treatment reduced shortening amplitudes only in WT-cells (plots averages of n = 5). **E-G**: Shortening-frequency plots of steady-state sarcomere shortening. Sarcomere shortening of WT- and TLR2-D cardiomyocytes was recorded within 60 min after isolation (Tyrode) or 6 h after incubation with LTA or in CM alone (**E, F**; * LTA vs. Tyrode, # LTA vs. CM). After 5 h of incubation with LTA in CM one group was transferred to CM with LTA supplemented with the iNOS-inhibitor SMT (LTA + SMT) (**G**; * LTA vs.CM, # LTA vs. LTA + SMT; **E-G**: n = 10–38 cells; mean ± SEM).

C. *In vitro cardiac function:* Starting at 1 up to 8 h after incubation with LTA (10 μg/ml) sarcomere shortening was recorded in isolated cardiomyocytes from WT and TLR2-D mice. Additionally, another subgroup of WT cardiomyocytes was incubated with LTA (10 μg/ml) for 6 h and the iNOS inhibitor S-methylisothiourea (SMT, 100 μM) was added after 5 h (Figure 5).

**Figure 6:**
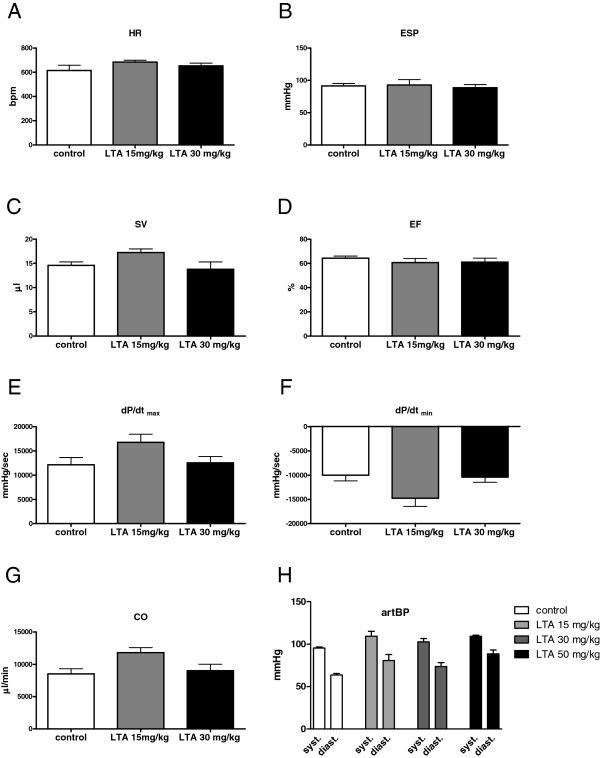
**Hemodynamic parameters in WT- and LTA-treated mice. A-G**: Cardiovascular function as measured with a pressure-volume catheter 4 h after LTA stimulation (15 or 30 mg/kg i.p.). LTA challenge did not influence hemodynamic parameters at this time point compared to control (HR = heart rate, ESP = end-systolic pressure, SV = stroke volume, EF = ejection fraction, first derivative of pressure rise = d*P*/dt_max_, first derivative of pressure fall = d*P*/dt_min_, CO = cardiac output; n = 6; mean ± SEM). **H**: Control recordings of arterial blood pressure (artBP) performed with a pressure catheter 6 h after LTA stimulation (15, 30 or 50 mg/kg i.p.). No influence on blood pressure after LTA challenge was observed (systolic arterial blood pressure (syst.); diastolic arterial blood pressure (diast.); n = 6; mean ± SEM).

D. *In vivo cardiovascular function:* 4 h after stimulation with different concentrations of LTA (15 and 30 mg/kg) hemodynamic parameters were monitored with a pressure-volume catheter in WT mice. For further corroboration, control experiments involving systemic invasive blood pressure measurements were performed in another study in WT animals with a pressure-catheter 6 h after stimulation with different concentrations of LTA (15, 30 and 50 mg/kg) (Figure 6).

### Stimulation protocol

Lipoteichoic acid (charge MGM5-10) was prepared as described previously [12]. Lipopolysaccharide (LPS) contamination was determined to be less than 1 EU/mg as determined by LIMULUS amoebocyte lysate assay (Charles River, Charleston, SC). Additionally, commercially available and highly purified LTA was purchased from Sigma-Aldrich (Sigma-Aldrich, Munich, Germany). These LTAs were applied in experiments of type A)-D).

In experiment type B) LPS (LPS; E. coli 0:111, Sigma Aldrich) and endotoxin-free cytidine triphosphate oligodesoxynucleotide (CpG-ODN; 1668-thioate; Tib-MolBiol, Berlin, Germany) [13] were applied alternatively. Purified LTA (15 mg/kg), LPS (20 mg/kg) and CpG-ODN (1 nmol/g 1668 thioate) were dissolved in endotoxin-free water. Previously, in a fibroblast assay we demonstrated that the chosen concentrations of LTA (15 mg/kg i.p.), LPS (20 mg/kg i.p.) and CpG-ODN (1 nmol/g i.p.) are equipotent with respect to TNF-activity induction [14].

Furthermore, LTA was tested in higher concentrations (30 mg/kg ≈ 600 μg/mouse; 50 mg/kg ≈ 1000 μg/mouse) for hemodynamic measurements in experiment type D). In all experiments each TLR-ligand was injected i.p. according to the study design before the animals were sacrificed.

### EMSA

Myocardial protein extracts were prepared with NE-PER^®^ Nuclear and Cytoplasmic Extraction Reagents (Perbio Science, Bonn, Germany), as previously published [13]. Nuclear factor 'kappa-light-chain-enhancer’ of activated B-cell (NFκB) oligonucleotides were end-labeled with Gamma 32P [γ-^32^P] adenosine triphosphate (ATP). Binding reactions (25 μl total) were performed with nuclear extracts. The specificity of DNA-protein binding was determined by cold chase (cc) analysis and supershift assays. Nuclear extracts were incubated with 2 mg of polyclonal anti-p50 (sc-114x) or anti-p65 (sc-109x) antibody (Santa Cruz Biotechnology, Santa Cruz, CA, USA). Deoxyribonucleic acid -protein complexes were electrophoresed; gels were dried, exposed overnight and scanned with a phosphoimager.

### mRNA isolation and RT-qPCR

Total ribonucleic acid (RNA) from whole hearts was isolated with the guanidinum thiocyanate method [6]. First-strand cDNA was synthesized according to the manufacturer’s protocol using the High-Capacity cDNA transcription kit (Applied Biosystems, Darmstadt, Germany) with random hexameric primers. Relative RT-qPCR was performed and analyzed with cDNA (diluted 1:10) on an ABI Prism 7900 Sequence Detection System and SDS2.2 Software (Applied Biosystems) using TaQman Gene expression Master Mix (part 4369016; Applied Biosystems) with the following primers: TLR2 (Mm01213946_g1), TNF-α (Mm00443258_m1), IL-1β (Mm99999061_mH) and the house-keeping gene GAPDH (Mm99999915_g1). All murine primers were measured using FAM TAMRA chemistry and the relative standard curve method. At the end of RT-qPCR cycle dissociation curve analysis was performed to ascertain the amplification of a single PCR product. Target gene expression was normalized to an internal control (glyceraldehyde-3-phosphate dehydrogenase, GAPDH) and relative quotient results were analyzed with GraphPad Prism 5 (GraphPad Software, San Diego, CA, USA).

### Enzyme linked immunosorbent assay

Whole hearts were dispersed with an Ultra-Turrax, homogenized and incubated on ice for 5 min in 1 ml of enzyme linked immunosorbent assay (ELISA) buffer containing phosphate buffered saline (PBS), Triton X-100 (1 μl/ml), phenylmethanesulfonyl fluoride (PMSF; 250 mM in isopropanol, 1 μl/ml) and protease inhibitors (Roche, Mannheim, Germany). Samples were incubated on ice for 20 min, vortexed and centrifuged for 15 min at 4°C. TNF-α and IL-1β protein levels were determined in the supernatant via ELISA (R&D Systems, Minneapolis, MN, USA).

### Ribonuclease protection assay

Cytokine messenger RNA (mRNA) levels were analyzed using a ribonuclease protection assay (RPA) for comparison of specific stimulation of TLR2, -4 and -9 4 h after application of the respective TLR ligand. Flash-frozen tissue was homogenized and total RNA was extracted by the guanidinium-thiocyanate method as described in [6].

The mRNA expression of TLR2, TLR4 and of pro-inflammatory mediators (TNF-α, IL-1β, IL-6, and iNOS) was determined with custom made template sets (BD Biosciences, Heidelberg, Germany). Signals were quantified with AIDA software v3.5 (Raytest, Straubenhardt, Germany) and normalized to the housekeeping gene ribosomal protein L32.

### Multiplex cytokine assay

Blood was obtained from the abdominal aorta 0, 2, 4 and 6 h after stimulation with LTA using heparinized syringes, centrifuged and the supernatant (serum) was stored at -20°C. After thawing, samples were analyzed immediately. Levels of TNF-α, IL-1β, IL-6 and IL-12 (Mouse cytokine multi-Plex for Luminex™ laser, BioSource Europe, Nivelles, Belgium) were determined using the microsphere array technique (Luminex Corporation, Austin, TX, USA) as previously described [15].

### Isolation, cell culture and incubation of cardiac myocytes

Excised hearts were prepared in Tyrode’s solution with ethylene glycol tetraacetic acid (EGTA) instead of CaCl_2_ (in mM: 135 NaCl, 4 KCl, 1 MgCl_2_, 2 hydroxyethyl piperazineethanesulfonic acid (HEPES), 2.6 EGTA, 10 glucose, and 1 mg/ml bovine serum albumin (BSA), pH 7.4) and mounted in a Langendorff perfusion system. Pressure was adjusted to 0.05 bar and the temperature to 36°C. Hearts were then perfused with the preparation solution for 5 min followed by a high-K^+^ solution for 5 min (in mM: 4 NaCl, 10 KCl, 130 K-glutamate, 1 MgCl_2_, 0.05 CaCl_2_, 2 HEPES, 10 glucose and 1 mg/ml BSA; pH 7.4 (KOH)). Trypsin (1000 BAE units/40 ml; Roche, Mannheim, Germany) and collagenase (type L, 25 mg in 40 ml, Sigma, St. Louis, MO, USA) were added to the high-K^+^ solution and hearts were perfused for 8–10 min in trypsin and for another 10–13 min in collagenase. Hearts were then sectioned into small parts and transferred into Tyrode’s solution, with 1.8 mM CaCl_2_ in place of EGTA and supplemented with trypsin-inhibitor 0.17 mg/ml (Sigma). The pieces were disintegrated by stirring with glass rods, and the solution was filtered and centrifuged. Cardiac myocytes of both mouse strains were tested immediately after isolation to determine baseline contractility. Treatment groups were incubated in culture medium (CM) consisting of Dulbecco’s modified Eagle medium, 5% minimal essential medium, 10% fetal calf serum, 50 μg/ml gentamicin (culture media from Gibco, New York, NY, USA) both with and without LTA (10 μg/ml) [16] from 1–8 h. Furthermore, the specific iNOS-inhibitor S-methylisothiourea (SMT; 100 μM; Sigma Aldrich) was added to the myocytes in culture one hour before measurement to investigate NO dependency of cardiac depression.

### Sarcomere shortening

Sarcomere shortening of isolated ventricular myocytes was recorded via a video imaging system and SarcLen^®^ software (IonOptix Limited, Milton, MA, USA) according to study design step C). The regular striation pattern of sarcomeres in a field of interest was analyzed using fast Fourier transformation (FFT). The video system was mounted onto an inverted microscope (Zeiss Axiovert 135TV, Jena, Germany, lens Fluar 40x 1.3) equipped with an experimental chamber perfused with Tyrode’s solution (≈ 600 μl/min leading to an exchange rate of three times per minute in the 200 μl volume of the chamber) heated to 36°C. Sarcomere shortening was monitored in Tyrode’s solution to avoid direct effects of the incubation media on the contractile response. Contractions were induced by bipolar external stimuli (0.4 ms, 30 V, SD9, Grass, Quincy, MA, USA). Stimuli were applied in pulse trains of 20 stimuli interrupted by 30 s stimulation pauses. The stimulation protocol was: 0.5, 10, 1, 8, 2, 6, and 4 Hz. The five last shortening signals of each train were averaged to obtain representative shortening frequency relationships. The resulting signal was evaluated for the following parameters: amplitude of sarcomere shortening, maximal first derivative of sarcomere shortening, and re-lengthening. For the identification and evaluation of staircases, trains of 20 original recordings of sarcomere shortening of cardiomyocytes were averaged. The peak values were fitted using a double exponential function as previously published [17].

### Hemodynamic measurements

Animals underwent hemodynamic measurement via pressure-volume catheter 4 h after LTA stimulation according to study design step D). After preparation of the right carotid artery a 1.4 F pressure conductance catheter (SPR-839, Millar Instruments, Houston, TX, USA) was inserted and advanced into the left ventricle. Data were recorded with Chart 5.5.5 (AD Instruments, Spechbach, Germany) and later analyzed with PVAN 3.6 (Millar Instruments). Volume signals were received in arbitrary relative volume unit numbers. Calibration of the conductance signal was performed in two steps: a) a 10 μl bolus of a hypertonic (10%) saline solution was injected into the external jugular vein to determine parallel conductance at the end of baseline measurement and b) whole blood drawn from the heart at the end of experiments was used for cuvette calibration. For this blood was loaded into a series of six cylinders, the conductance of each was recorded, and a calibration curve was generated according to the manufacturer’s protocol. The following parameters were recorded: heart rate (HR); end systolic pressure (ESP); stroke volume (SV), ejection fraction (EF); maximal first derivative of pressure rise (d*P*/dt_max_), maximal first derivative of pressure fall (d*P*/dt_min_) and cardiac output (CO).

In an additional set of independent control experiments hemodynamic parameters were recorded by a second investigator with a 1.4 F pressure catheter (Millar instruments, Houston, TX, USA) instead of the pressure-volume catheter. The following parameters were measured: HR; systolic arterial pressure (SAP); diastolic arterial pressure (DAP; not shown); left ventricular systolic pressure (LVSP; not shown), left ventricular end-diastolic pressure (LVEDP; not shown); d*P*/dt_max_, d*P*/dt_min_ (not shown). Furthermore, in this series of experiments LTA from Sigma-Aldrich was applied (15, 30, 50 mg/kg) for 6 h.

### Statistical analysis methods

All values are expressed as mean ± SEM. One-way ANOVA analyses followed by Newman-Keuls-Tests *post hoc* analysis were used to determine significant differences using GraphPad Prism 5.0. Differences were considered to be significant at p < 0.05.

## Results

### Clinical symptoms after LTA challenge

Following 4 h of LTA challenge, WT-mice developed shock-like symptoms such as lethargy, nasal and ocular discharge as well as piloerection. Severity of these symptoms appeared to be associated with the applied LTA dosage. Toll like receptor 2-D mice did not show any clinical signs of sickness. Lipopolysaccharide or CpG-ODN challenge initiated signs of severe inflammation starting 2 h after stimulation [13].

### Cardiac TLR2 expression

Lipoteichoic acid induced a significant up-regulation of TLR2 mRNA in myocardial tissue after 2 h in comparison to baseline and to TLR2-D mice (Figure 1A). Peak expression was detected 4 h after stimulation (≈7-fold increase in WT). An apparent TLR2 increase in TLR2-D mice (not significant) might be linked to the RT-qPCR primer construct binding rudimentary parts of the C-terminal promotor region for TLR2 according to the manufacturer.

### NFκB activation in myocardial tissue

The time course of myocardial NFκB-DNA binding activity following LTA stimulation is depicted in Figure 1B. Lipoteichoic acid treatment led to robust time-dependent binding activity of myocardial NFκB in WT-mice starting at 1 h and lasting up to 4 h. In TLR2-D mice NFκB regulation was not detectable. The NFκB-complex mainly consisted of p50 and p65 as detected in the supershift assay.

### Myocardial cytokine mRNA and protein expression

Lipoteichoic acid induced an increase of TNF-α and IL-1β mRNA transcripts in hearts of WT-mice with a TNF-α peak at 4 h and an IL-1β peak at 2 h after injection of LTA (TNF-α: ≈ 14-fold and IL-1β: ≈ 22-fold in WT). In TLR2-D animals this effect could not be detected (Figure 2A-B).

Respective protein expressions in the heart were measured by ELISA (Figure 2C-D). Lipoteichoic acid administration led to an increase in protein expression of both TNF-α and IL-1β in WT-mice with a maximum at 4 h (TNF-α ≈ 3-fold and IL-1β ≈ 8-fold compared to control), which was not observed in TLR2-D animals.

Cytokine mRNA expression after stimulation with different TLR-ligands was detected by RPA. At 4 h, LTA stimulation resulted in an increase of mRNA expression of TLR2, TNF-α and IL-1β (Figure 4), which was in accordance with our results from RT-qPCR. Lipopolysaccharide stimulation resulted in a significant increase of all investigated parameters 4 h after application. CpG-ODN challenge led to a significant increase of TNF-α and TLR2.

### Serum cytokine levels

Multiplex cytokine assay revealed that LTA-treated WT animals showed an increase of TNF-α, IL-1β and IL-12 serum levels 2 and 4 h after stimulation (Figure 3A, B, D). Interleukin-6 however, was increased only 2 h after stimulation (Figure 3C). In TLR2-D mice, LTA stimulation did not induce any cytokine expression.

### Influence of LTA on sarcomere shortening

Murine cardiomyocytes exhibit a characteristic frequency-dependent shortening pattern after a stimulation rest. The post-rest shortening is followed by a negative staircase at low frequencies (Figure 5A-D) (< 6 Hz) and a positive staircase at high frequencies (> 6 Hz; data not shown). This contractility pattern was similar among cardiomyocytes from both WT and TLR2-D mice (Figure 5A-D). Treatment of cardiomyocytes with LTA (10 μg/ml) for more than 4 h depressed both post-rest and steady-state shortening of WT but not TLR2-D cardiomyocytes (Figure 5C, D). A detailed analysis exhibited a tendency to decreased shortenings 3 h after the start of treatment reaching significance at 4 h (data not shown).

Steady state shortening exhibits a biphasic shortening-frequency relationship negative below 2 Hz and positive above 2 Hz. At 2 Hz shortening was minimal (Figure 5E-G) in both WT and TLR2-D cells. Lipoteichoic acid induced a significant decrease of shortening amplitude in WT cells at 0.5, 4 and 6 Hz as compared to culture medium (CM) (Figure 5E). Lipoteichoic acid changed frequency-dependent shortening behavior such that the shortening amplitude was depressed to a higher degree at low frequencies than at high frequencies. At the highest frequency (10 Hz = physiological heart rate of mice) LTA did not depress the shortening amplitude. Steady-state shortening of TLR2-D cells was insensitive to LTA challenge (Figure 5F). The speed of sarcomere shortening as well as re-lengthening showed the same frequency relationship as the sarcomere shortening amplitude. In WT-cells LTA suppressed both parameters significantly (data not shown). To further elucidate a possible underlying mechanism we investigated the influence of iNOS inhibition on LTA-dependent suppression of sarcomere shortening (Figure 5G). Again, the steady-state shortening amplitude was significantly reduced at 0.5, 1, 2, 4, 6 and 8 Hz. Addition of S-methylisothiourea (SMT, 100 μM) applied during the last hour of LTA treatment reversed the reduction of shortening completely (Figure 5G).

### Hemodynamic function after LTA challenge

Measurements with a pressure-volume catheter were performed 4 h after LTA stimulation because cytokine protein expression peaked at this time point. These recordings revealed that HR, ESP, SV, EF, d*P*/dt_max_, d*P*/dt_min_ and CO were not influenced after 4 h (Figure 6A-G). Even doubling of the LTA dosage (30 mg/kg, i.p.) did not alter cardiac function (Figure 6A-G). In a control experiment performed with a pressure catheter by a second investigator 6 h after LTA administration (15, 30 or 50 mg/kg) hemodynamic parameters were monitored. Interestingly, here again LTA did not influence most of the recorded hemodynamic parameters (SAP, DAP: Figure 6H; LVSP, LVEDP, d*P*/dt_max_, d*P*/dt_min_: data not shown) with the exception of HR, which was significantly elevated 6 h after application of 50 mg/kg LTA (data not shown).

## Discussion

This study shows the effects of highly purified LTA on cardiac inflammation and hemodynamic parameters *in vivo* for the first time. Lipoteichoic acid application induced an inflammatory reaction in the heart, which was reflected in an up-regulation of systemic cytokine levels. However, hemodynamic parameters *in vivo* were not decreased by LTA challenge. In contrast to these observations, *in vitro* incubation of cardiac myocytes with LTA reduced sarcomere shortening in an NO-dependent manner. As LTA induced clinical symptoms but hemodynamic changes seemed to be moderate we compared LTA inflammatory effects with those of TLR4 and TLR9 ligands.

The murine myocardium constitutively expresses TLR2 [9,18] but, so far, little is known about its role in septic myocardial impairment. We stimulated TLR2 with its specific ligand LTA [19], which led to a significant up-regulation of TLR2 gene expression peaking after 4 h. These findings support the previous observation that TLR2 up-regulation in the heart can also be induced by heat inactivated *S. aureus*[9]. Hence, it can be speculated that TLR2 induction plays an important role in long-lasting inflammation. Similarly, TLR2 up-regulation has been reported for other organs and under conditions considerably different from ours [20,21].

Our study revealed robust activation of NFκB following LTA stimulation in the heart, which ultimately resulted in an increased release of TNF-α and IL-1β. This effect was not observed in TLR2-D animals. Another TLR2 ligand, peptidoglycan-associated lipoprotein, also increased TNF-α expression in HL-1 cardiomyocytes in the study of Zhu et al. [22]. In addition, in whole rat hearts mounted on a Langendorff perfusion TNF-α levels increased after LTA application [16].

In a fibroblast assay we demonstrated earlier that the applied concentrations of LTA (15 mg/kg i.p.), LPS (20 mg/kg i.p.) and CpG-ODN (1 nmol/g i.p.) are equipotent with respect to TNF-activity induction [14]. These equivalent concentrations of LPS, LTA and CpG-ODN were applied in this experimental setting as well. Consequently, all three stimuli significantly induced TNF-α expression in the heart. Unlike TNF-α, other inflammatory mediators were differentially regulated by the three TLR-ligands. The strongest induction was caused by LPS, which elevated levels of all tested mediators significantly. As LPS and CpG-ODN have been shown to impair whole heart contractility *in vivo*[23,24] and cardiomyocyte shortening [13,25], we challenged isolated cardiomyocytes with 10 μg/ml of LTA and recorded sarcomere shortenings. This *in vitro* applied concentration of LTA is well in the range of earlier investigations. Grandel et al. demonstrated cardiac depression in an *ex vivo* Langendorff perfusion at 2 and 10 μg/ml of LTA [16]. Furthermore, 10 μg/ml of LTA has been shown to markedly inhibit neutrophil chemotaxis [26]. In the present study LTA challenge induced a significant depression of cardiomyocyte sarcomere shortening at stimulation frequencies ≤ 8 Hz, which was not observed in TLR2-D cells. This is in agreement with a previous study by et Zhu al., in which peptidoglycan-associated lipoprotein diminished both calcium transients and sarcomere shortenings in cardiomyocytes in a TLR2- and MyD88-dependent manner at 1 Hz [22]. It should be noted that a stimulation frequency of 1 Hz corresponds to a heart frequency of 60 bpm and hence, is not relevant *in vivo* in mice. To simulate *in vivo* heart frequencies in mice isolated cardiomyocytes were stimulated with frequencies up to 10 Hz in this experiment. Interestingly, the extent of depression weakened at stimulation frequencies above 6 Hz and was completely absent at 10 Hz, i.e. LTA did not reduce sarcomere shortening at the physiological heart rate of mice. In contrast it has been shown earlier that LPS and CpG-ODN diminished sarcomere shortening independently of the stimulation frequency up to 10 Hz [13,25]. We found depression of sarcomere shortening occurred after 4 h of LTA incubation, which paralleled the peak TNF-α protein levels *in vivo*. This is supported further by the observation of Grandel et al. that LTA induced whole heart cardiac depression could be prevented by application of a neutralizing TNF-α antibody [16]. In addition, it has been demonstrated that TNF-α initiates excessive NO release either via cardiac endothelial nitric-oxide synthase (eNOS) or via iNOS [7]. In our *in vitro* experiments the application of the highly selective iNOS-inhibitor SMT abolished the suppression of sarcomere shortening indicating an NO-dependency. Nitrous oxide-dependency of depressed sarcomere shortening was observed earlier after LPS and CpG-ODN challenge of isolated cardiomyocytes [13,25].

To further elucidate the influence of LTA stimulation on cardiac function *in vivo* we measured hemodynamic parameters with a pressure-volume catheter. Surprisingly, no alteration of myocardial function was observed after challenge with LTA (15 mg/kg i.p.) *in vivo*. Even after increasing the dosage to 30 mg/kg, LTA did not affect contractile function. To further confirm these results, a second experiment was conducted using a pressure catheter and commercially available LTA charges. Again LTA did not influence hemodynamic parameters. Therefore, the LTA dosage as well as the exposure time was increased to 50 mg/kg and 6 h. Unexpectedly, HR increased under these conditions.

The surprising difference between the *in vitro* and *in vivo* effects on cardiac activity might be explained by differences in the concentration of LTA in both situations. However, 10 μg/ml *in vitro* equals 10 mg/kg, which is in the range of the *in vivo* dosage applied in this study. This LTA dosage was much higher than the one used by Finney et al. showing changes in leukocyte adhesion (50 μg/kg estimated from 100 μg/rat) [27] and the 4 mg/kg used by Zhao et al. [28]. Others have applied between 10–1000 μg/mouse LTA, thereby inducing a distinct microRNA pattern [29]. Thus, the dosage of LTA applied here was in the range of the highest doses found in literature, provoked cardiac inflammation and should have also been able to cause consecutive depressed cardiac function. However, this latter effect was not observed *in vivo*.

To explain this surprising finding the experiments on isolated cardiomyocytes should be taken into account in detail. Isolated cardiomyocytes were incubated with LTA in culture dishes and attachment of LTA to polystyrene has been shown to augment its immunostimulatory potency, which could be abrogated in the presence of plasma [30]. However, in our experiments cardiomyocytes were incubated with LTA in the presence of 10% fetal calf serum, which may share some components with plasma. Therefore, this surface-dependent augmentation of LTA effects may have contributed to, but cannot fully explain the differences seen between *in vitro* and *in vivo* experiments. Furthermore, *in vitro* we found depression of sarcomere shortening only at stimulation frequencies ≤ 8 Hz, which is clearly below the physiological heart rate of mice. In earlier experiments using heat-inactivated *S. aureus* as a stimulus cardiac contractility decreased, as measured in a Langendorff preparation [9]. But stimulation frequency was also below the physiological range in these experiments. In addition, the immunological potency of heat-inactivated *S. aureus* LTA may be higher than that of purified LTA, as LTA lacking lipoproteins has been shown to be less potent than WT LTA due to different signaling mechanisms [31].

Reduced sarcomere shortening *in vitro* could be antagonized by an iNOS inhibitor (SMT). In contrast, increased iNOS expression after LTA treatment in the heart *in vivo* could not be detected here nor in rat heart by others [16]. But, significant iNOS elevation has been reported due to the simultaneous application of LTA and peptidoglycan (PGN) [32]. Recently PGN and its subcomponent muramyl dipeptide (MDP) have been shown to signal only via NOD receptors [33,34] indicating a more complex synergistic mechanism. Furthermore, some inflammatory responses initially associated with specific TLR2-ligand-derived signaling have been attributed to LPS contamination later [35].

Vascular contractility is known to contribute decisively to maintenance of arterial blood pressure. Thus, the sensitivity of the arterial wall to LTA will influence the blood pressure in the situation of a TLR2-dependent inflammation. Recently it has been shown that LTA application to isolated blood vessels can even improve vascular contractility [11]. This effect may support the stability of SABP observed here.

## Conclusions

Toll-like receptor 2-stimulation *in vivo* with LTA alone induced only a moderate inflammatory response compared to stimulation via TLR4 or TLR9. One might speculate that Gram-positive bacteria initiate innate immunity to a greater extent when diverse PAMPS and antigens of the bacterium are presented and thus, the numerous receptor-signaling networks of innate immunity are induced in close cooperation. LTA alone displays moderate pro-inflammatory properties via TLR2 alone, LTA and PGN show a strong synergistic effect via TLR2 and NOD2 [33] and whole bacteria might additionally stimulate TLR9 via their bacterial DNA [13]. These findings indicate that purified LTA contributes to cardiac inflammation, yet does not significantly influence cardiac function. Given the intricacy of innate immunity, the role of LTA cannot yet be resolved. Future detailed studies of single TLR-dependent immune responses are required to demonstrate the extent to which LTA is relevant in the clinical context.

## Competing interests

The authors declare that they have no competing interests.

## Authors’ contributions

OB: animal experiments, drafting of the manuscript, interpretation and statistical analysis of the data, PK: animals experiments, conceived of the study, wrote the grant application, drafting of the manuscript, interpretation of the data, JP: sarcomere shortening, MS: RT-qPCR, HE: RPA, ELISA. SK: invasive pressure catheter measurements, RL: invasive pressure-volume catheter measurements, MV: EMSA for NFκB, SM: preparation and distribution of highly purified LTA, testing for LPS contamination, AK: Luminex analysis of blood plasma, KZ: Luminex analysis of blood plasma, CG: drafting of the manuscript, AH: drafting of the manuscript, GB: supervision of the lab, drafting of the manuscript, RM: conceived the study, drafting of the manuscript, interpretation of the data. All authors read and approved the final manuscript.
